# Concurrent pathologic femoral shaft fracture in bone metastasis and acute myocardial infarction: a case report

**DOI:** 10.1093/jscr/rjaf1108

**Published:** 2026-01-20

**Authors:** Yusei Katsuyama, Shinichiro Nakamura, Kentaro Sasaki, Tomoki Saito, Kenji Takahashi

**Affiliations:** Department of Orthopedics, Graduate School of Medical Science, Kyoto Prefectural University of Medicine, 465 Kajii-cho, Kamigyo-ku, Kyoto 602-8566, Japan; Department of Orthopaedics, Fukuchiyama City Hospital, 231 Atsunaka-cho, Fukuchiyama, Kyoto 620-8505, Japan; Department of Orthopaedics, Fukuchiyama City Hospital, 231 Atsunaka-cho, Fukuchiyama, Kyoto 620-8505, Japan; Department of Orthopedics, Graduate School of Medical Science, Kyoto Prefectural University of Medicine, 465 Kajii-cho, Kamigyo-ku, Kyoto 602-8566, Japan; Department of Orthopedics, Graduate School of Medical Science, Kyoto Prefectural University of Medicine, 465 Kajii-cho, Kamigyo-ku, Kyoto 602-8566, Japan

**Keywords:** hepatocellular carcinoma, pathological femoral fracture, bone metastasis, acute myocardial infarction, embolization, percutaneous coronary intervention

## Abstract

An 86-year-old woman with a history of hepatocellular carcinoma was brought to our emergency department after an indoor fall. Her chief complaint was pain in the left thigh and chest. The patient was diagnosed with a pathological femoral shaft fracture, bone metastasis, and acute myocardial infarction. She underwent preoperative embolization and percutaneous coronary intervention, followed by internal fixation on the same day as the injury. The patient was discharged without postoperative myocardial infarction and was able to walk independently using a walker. Femoral diaphyseal fractures and coronary events require urgent intervention, and their coexistence makes it particularly difficult to determine the treatment sequence and timing. In addition, if the fracture is pathological due to bone metastasis, management becomes even more challenging. A multidisciplinary team is essential for the successful management of such complex cases.

## Introduction

Acute coronary syndromes are emergent conditions that often require immediate coronary intervention [1]. Patients undergoing percutaneous coronary intervention (PCI) for acute myocardial infarction (AMI) may require non-cardiac surgery (NCS) to minimize perioperative cardiovascular events [1]. However, femoral shaft fractures require urgent attention because treatment delays can increase mortality [2, 3]. The coexistence of these conditions makes management increasingly challenging, and pathological fractures from bone metastases are even more difficult to treat.

The optimal sequence and timing of treatment for such cases remain unclear. Herein, we report a case of a pathological femoral shaft fracture due to bone metastasis with concurrent AMI.

## Case report

An 86-year-old Japanese woman fell indoors and was transported to the emergency department by ambulance. She complained of pain in the left thigh and chest. Her medical history included hepatocellular carcinoma (HCC) diagnosed a year earlier, treated with transcatheter arterial chemoembolization and radiation therapy, achieving a complete response. She had no coronary risk factors, including hypertension, hyperlipidaemia, diabetes, or smoking.

Her vital signs were: heart rate, 77 beats/min; blood pressure, 143/80 mmHg; respiratory rate, 15 breaths/min; and oxygen saturation, 99% on room air.

**Figure 1:**
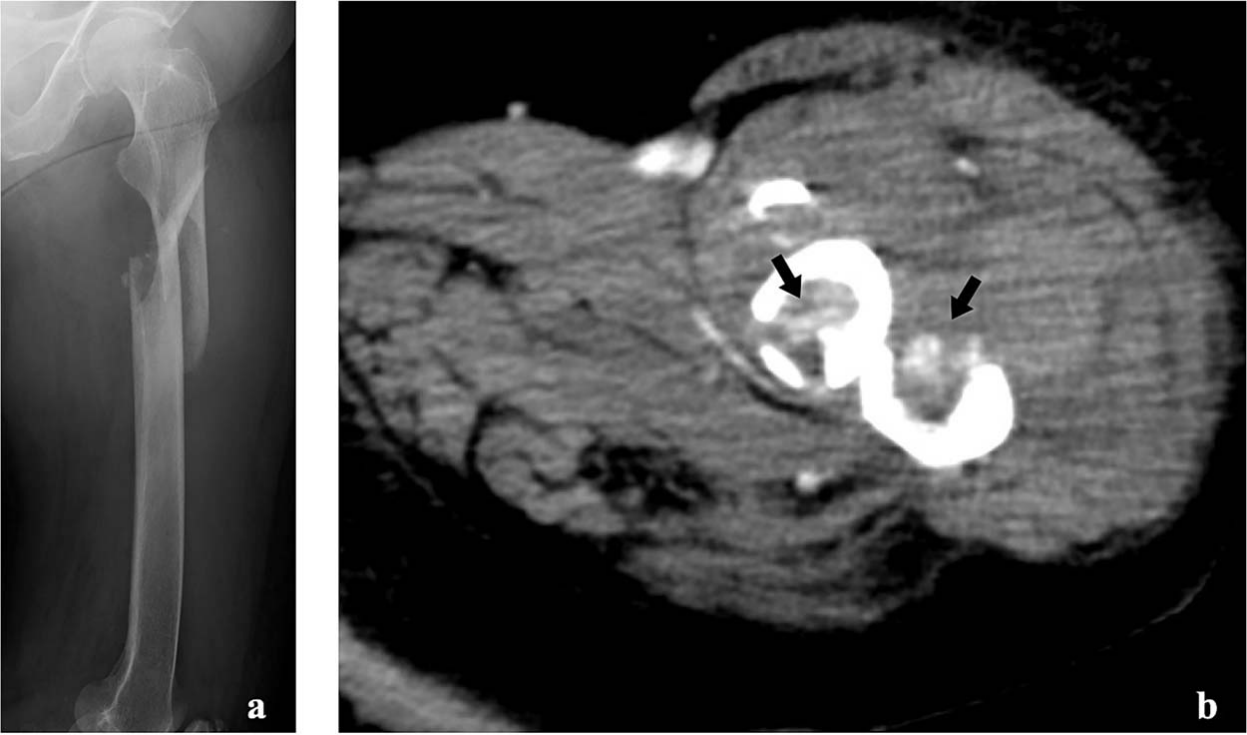
Imaging tests of the left femur. (a) Radiograph showing a fracture of the left femoral shaft. (b) Contrast-enhanced computed tomography showing the tumour strain around the fracture site.

Radiography revealed a femoral shaft fracture (Fig. 1a), and contrast-enhanced computed tomography revealed a blush around the fracture site (Fig. 1b). Electrocardiography indicated sinus rhythm with ST depression in V3–V6. Echocardiography revealed severe anteroseptal hypokinesis and left ventricular ejection fraction <40%.

Laboratory tests showed troponin I level of 6.63 ng/ml (normal < 0.04), creatine kinase MB 76 U/l (normal < 25), white blood cell 7190/μl (normal 3500–8500), haemoglobin 7.0 g/dl (normal 11.7–15.6), and platelets 9.6 × 10^4^/μl (normal value: 14–34 × 10^4^).

**Figure 2:**
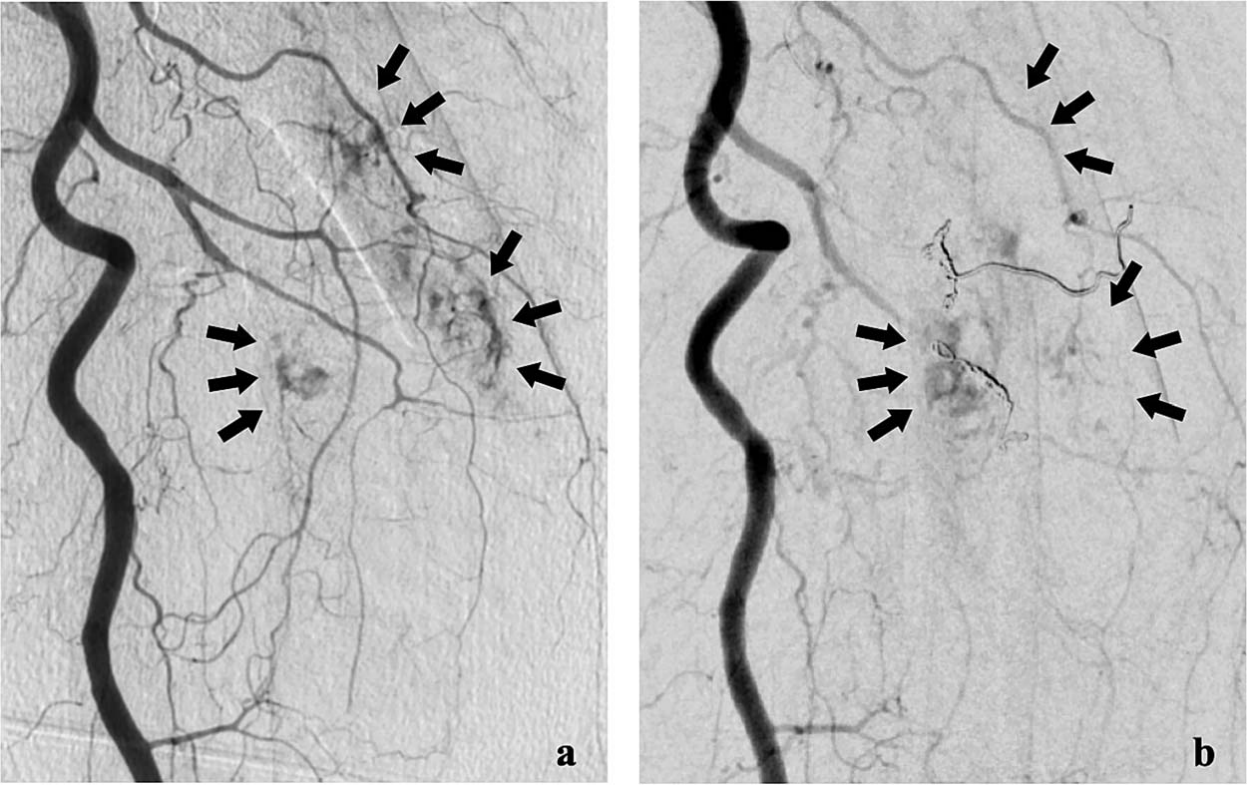
Lower extremity angiography. (a) Pre-embolization angiography showing tumour blush in the femur. (b) Post-embolization angiography showing tumour devascularization.

**Figure 3:**
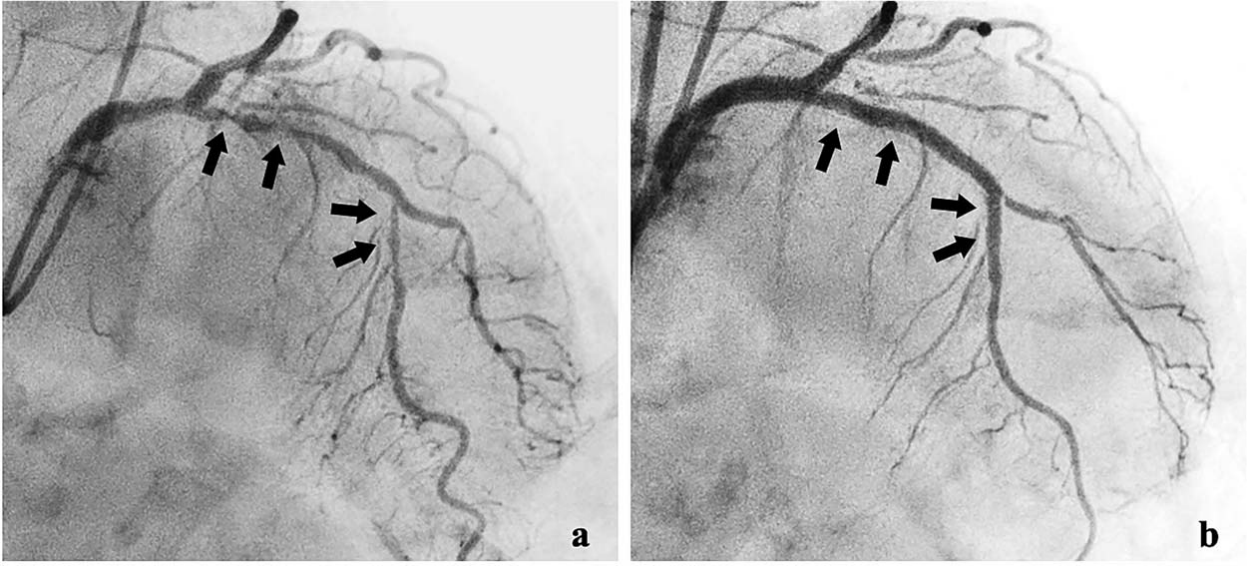
Coronary angiography findings. (a) Pre-treatment angiography showing 90% stenosis of the left anterior descending artery. (b) Post-treatment angiography showing improved blood flow in the left anterior descending artery after percutaneous coronary intervention.

She was transferred to the catheterization laboratory, where coronary and lower extremity angiography revealed 90% stenosis of the left anterior descending artery and tumour blood supply via branches of the deep femoral artery (Figs 2a and 3a).

She was diagnosed with a pathological femoral shaft fracture from bone metastasis and non-ST-segment elevation myocardial infarction. The revised Katagiri score was 5, and the Charlson comorbidity index was 12.

After a multidisciplinary team (MDT) review, transcatheter arterial embolization was initially performed for the bone metastasis. The tumour received blood from two deep femoral artery branches, and embolization with gelatin sponges and coils achieved adequate devascularization (Fig. 2b).

Dual antiplatelet therapy (DAPT) with aspirin and prasugrel was initiated, followed by PCI with drug-eluting stents (DES) from the left main coronary trunk into the anterior descending artery (Fig. 3b).

**Figure 4:**
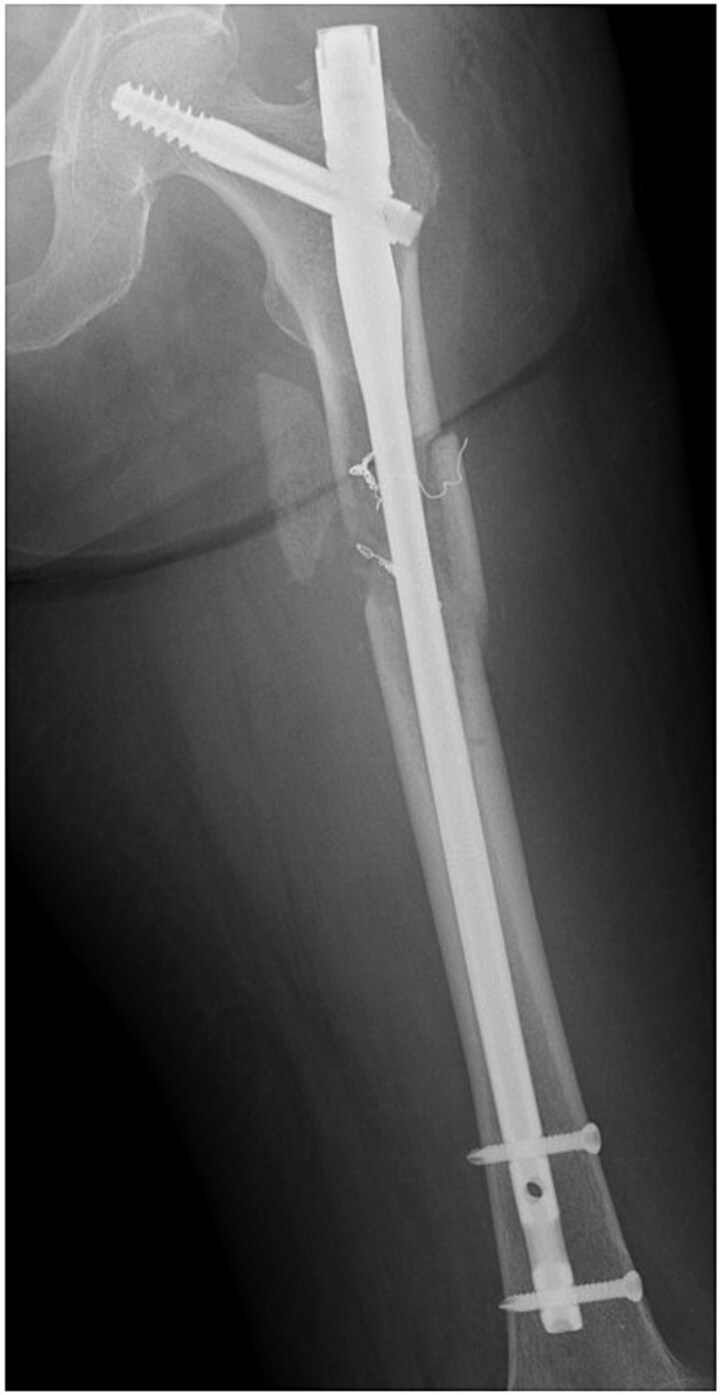
Postoperative radiograph of the left femur.

After interventional radiology, internal fixation with antegrade intramedullary nailing was performed using the blocking pin technique (Fig. 4). Tumour tissue from the fracture site was sent for pathology. The operative time was 63 min, and intraoperative blood loss (IBL) was 30 ml. She was allowed full weight-bearing depending on her condition.

Postoperatively, she was admitted to the intensive care unit (ICU), where emergency physicians and cardiologists managed her care. She developed acute heart failure requiring diuretics but no postoperative myocardial infarction. After 7 days, she was discharged from the ICU in stable condition. Pathology confirmed bone metastasis from HCC. Postoperative femoral radiation therapy and subcutaneous denosumab were administered. She regained mobility with a walker and was discharged 50 days after admission, remaining free of complications at 1-year follow-up.

## Discussion

In patients with confirmed AMI, current guidelines prioritize prompt coronary revascularization and recommend postponing NCS, as perioperative major adverse cardiovascular events (MACEs) decrease with longer intervals between PCI and surgery [1]. When surgery cannot be safely deferred, it is reasonable to proceed with careful haemostatic planning while continuing antiplatelet therapy, as appropriate [1]. NCS should typically be scheduled after PCI as follows: ~12 months after DES placement for acute coronary syndrome and at least 6 months after DES placement for chronic coronary disease. Time-sensitive surgery may be considered after 3 months if the risk of delay exceeds cardiac risk [1]. If antiplatelet interruption is unavoidable during urgent surgery, balloon angioplasty without stenting may be used, with surgery deferred for at least 14 days [4]. Surgery within 30 days of PCI carries a high risk of MACEs, and DAPT should not be stopped early because of stent thrombosis [1].

Femoral shaft fractures have better outcomes with early fixation; delays beyond 24–48 h are associated with increased morbidity and mortality, particularly in older adults [3]. However, mortality and complication rates for pathologic hip fractures due to bone metastases reportedly do not differ whether surgery occurs within 2 days or later [5]. Planning treatment for such patients is complex, and coordination across teams requires time. Surgery should not be unnecessarily delayed; however, if additional time benefits the patient, the procedure should not be hastened.

In this case, as HCC is a hypervascular tumour and the patient's haemoglobin level at admission was low (7.0 g/dl), and since DAPT increases bleeding risk, we judged that the disadvantages of delay were significant. We therefore performed preoperative embolization to control haemorrhage and reduce IBL, followed by DAPT initiation and PCI. Preoperative embolization can reduce IBL without significant complications [6] and is most effective when performed within a day before surgery [7].

The treatment strategy for pathological femoral fractures varies according to the patient's condition and prognosis [8]. This patient had a revised Katagiri score of 5, placing her in the intermediate-risk group with a predicted one-year survival rate of ~50% [9]. Although radical treatment for femoral bone metastasis could have been considered, palliative surgery was preferred due to poor general health. Closed reduction was performed using the Poller pin technique and internal fixation with an intramedullary nail. The blocking screw technique reportedly increases bone union rates while reducing IBL, complication rates, and hospital stay [10].

This case illustrates a rare pathological femoral diaphyseal fracture from HCC metastasis with concurrent AMI, managed with same-day embolization, PCI with DAPT, and minimally invasive intramedullary nailing. The MDT balanced two time-sensitive priorities: coronary revascularization for AMI and early fracture stabilization in older patients.

In conclusion, multidisciplinary, well-coordinated management may be crucial for patients with bone metastases and acute coronary events.

## Data Availability

The datasets used and analysed in the current study are available from the corresponding author upon reasonable request.
